# Functional Characterization and Drug Response of Freshly Established Patient-Derived Tumor Models with CpG Island Methylator Phenotype

**DOI:** 10.1371/journal.pone.0143194

**Published:** 2015-11-30

**Authors:** Claudia Maletzki, Maja Huehns, Patrick Knapp, Nancy Waukosin, Ernst Klar, Friedrich Prall, Michael Linnebacher

**Affiliations:** 1 Molecular Oncology and Immunotherapy, University of Rostock, Rostock, Germany; 2 Institute of Pathology, University of Rostock, Rostock, Germany; 3 Department of General Surgery, University of Rostock, Rostock, Germany; Sapporo Medical University, JAPAN

## Abstract

Patient-individual tumor models constitute a powerful platform for basic and translational analyses both *in vitro* and *in vivo*. However, due to the labor-intensive and highly time-consuming process, only few well-characterized patient-derived cell lines and/or corresponding xenografts exist. In this study, we describe successful generation and functional analysis of novel tumor models from patients with sporadic primary colorectal carcinomas (CRC) showing CpG island methylator phenotype (CIMP). Initial DNA fingerprint analysis confirmed identity with the patient in all four cases. These freshly established cells showed characteristic features associated with the CIMP-phenotype (HROC40: APC^wt^, TP53^mut^, KRAS^mut^; 3/8 marker methylated; HROC43: APC^mut^, TP53^mut^, KRAS^mut^; 4/8 marker methylated; HROC60: APC^wt^, TP53^mut^, KRAS^wt^; 4/8 marker methylated; HROC183: APC^mut^, TP53^mut^, KRAS^mut^; 6/8 marker methylated). Cell lines were of epithelial origin (EpCAM^+^) with distinct morphology and growth kinetics. Response to chemotherapeutics was quite individual between cells, with stage I-derived cell line HROC60 being most susceptible towards standard clinically approved chemotherapeutics (e.g. 5-FU, Irinotecan). Of note, most cell lines were sensitive towards “non-classical” CRC standard drugs (sensitivity: Gemcitabin > Rapamycin > Nilotinib). This comprehensive analysis of tumor biology, genetic alterations and assessment of chemosensitivity towards a broad range of (chemo-) therapeutics helps bringing forward the concept of personalized tumor therapy.

## Introduction

Three main molecular pathways have been recognized in colorectal cancer (CRC). These include I) chromosomal instability (CIN); II) microsatellite instability (MSI), and III) CpG island methylator phenotype (CIMP). The latter subtype being first formulated by Toyota et al. [1], is defined by aberrant DNA methylation, leading to concordant promoter hypermethylation of multiple genes. CpG island DNA methylation occurs early in malignant transformation [2] and consequently results in silencing of normal tumor-suppressor function and cancer formation, independent of (physiological) age-related methylation. The CIMP subtype exists with and without MSI, both showing distinct morphologic, molecular and most importantly, clinical characteristics [3]. MSI-high/CIMP^+^ tumors, related to BRAF^V600E^ mutations and MLH1 promoter methylation, are usually associated with low cancer-specific mortality, mostly due to their natural immunogenicity [3]. By contrast, CIMP without MSI is independently related to significantly worse outcome [3–5]. The unique molecular signature includes a high frequency of KRAS mutations (>60%) and usually less extensive promoter methylation (designated as "CIMP-Low” vs. CIMP-High in MSI^+^ tumors) [6–8].

Determining the precise genetic and/or epigenetic alterations of each cancer case is important to defining treatment strategies and predicting prognosis. To date, chemotherapy response of CIMP^+^ tumors is still controversial. Preceding studies reported different results on 5-FU-based therapy, ranging from good response up to complete resistance [9, 10]. Very recently, methylation inhibitors have become increasingly recognized as another option for treatment of CIMP^+^ tumors and hence, a clinical phase I/II trial has just been initiated (trial number: NCT01193517). In this study, metastatic colorectal cancer patients are being treated with Azacytidine (5-Aza-2-deoxycytidine) and CAPOX (Capecitabine + Oxaliplatin). First results will be published in 2016.

Additionally to attempts in improving treatment options, discovery of novel diagnostic and/or prognostic CRC-specific (DNA methylation) markers is ongoing [11]. To reflect the complexity and diversity of CIMP^+^ tumors, both approaches may ideally be performed in patient-individual tumor models. Here, such models generated from four individual CIMP^+^ tumors (CIMP-L and CIMP-H) are presented.

## Material and Methods

### Tumor preparation and cell line establishment protocol

Primary CRC cases were obtained upon resection, with informed written patient consent (n = 4). All procedures were approved by the institutional Ethics Committee (Ethikkommission an der Medizinischen Fakultät der Universität Rostock; St.-Georg-Str. 108, 18055 Rostock; reference number II HV 43/2004) in accordance with generally accepted guidelines for using human material. None of the patients’ received prior tumor-related therapy. Upon surgical removal, tumor samples were processed further as described [12, 13]. Briefly, pieces of tumors (3 x 3 x 3 mm) were frozen viable (FCS, 10% DMSO) at -80°C for subsequent xenografting into NMRI Foxn1^nu^ mice. Other pieces were stored in liquid nitrogen for molecular analysis. Cell culture was started from single cell suspensions, seeded on collagen-coated plates in complete tumor medium and incubated at 37°C in a humidified atmosphere of 5% C0_2_. All cell culture reagents were obtained from PAN Biotech (Aidenbach, Germany), antibiotics and antifungal agents were provided by the university hospital’s pharmacy. Continually growing cell cultures were serially passaged and regularly stocked in low passages.

For *in vivo* engraftment, five to six-week-old female NMRI Foxn1^nu^ mice were used as recipients. Mice were bred in the university’s animal facility and maintained in specified pathogen-free conditions in accordance with guidelines as put forth by the German Ethical Committee. All *in vivo* procedures were approved by the Committee on the Ethics of Animal Experiments of the University of Rostock (Landesamt für Landwirtschaft, Lebensmittelsicherheit und Fischerei Mecklenburg-Vorpommern; Thierfelder Str. 18, 18059 Rostock, Germany; approval number: LALLF M-V/TSD/7221.3–1.1-015-14). Subcutaneous (s.c.) tumor implantation into both flanks was performed under Ketamin/Xylazin anesthesia (dose: 90/6 mg/kg bw), and all efforts were made to minimize suffering. Established xenografts (≥1500 mm^3^) were removed and stored vitally for further analysis.

### Tumor histology and immunohistochemistry

Histopathological examination of the primaries was done according to standard protocols for clinicopathological CRC staging [14], and additional staging information was compiled from patients' clinical charts. H & E sections and β-catenin immunostainings were obtained from paraffin-embedded tumors.

### Molecular analysis

Molecular classification, MSI and mutational analysis of tumor-associated genes (APC, TP53, KRAS and B-Raf^V600E^), as well as DNA methylation in CIMP-sensitive promoters was done as described [12, 13, 15]. All data including staging information compiled from the clinical charts are summarized in Table 1. To classify CIMP, a combined panel covering eight markers was applied. Analyzed markers resulted from those originally described by [16, 17]. On a basis of this panel, tumors with 1–5/8 methylated promoters are being classified as CIMP-L and with 6–8/8 methylated promoters as CIMP-H [18]. Chromosomal instability (CIN) was assessed using SNP Array 6.0 from Affymetrix (Cleveland, OH) according to manufacturer’s instructions.

**Table 1:** Clinical and pathological characteristics of patients as well as cell line establishment protocol.

Tumor-ID	Age/ Gender	Tumor location	Grade and TNM-Stage	UICC Stage	β-Catenin translocation	Direct cell line establishment	Cell line from xenograft	Corresponding xenograft	Paired B-LCL
HROC40	69/m	descending colon	G3T3N1M0	IIIa	+	+	-	+	yes
HROC43	72/m	ascending colon	G3T3N2M0	IIIb	-	+	-	-	yes
HROC60	71/m	ascending colon	G2T2N0M0	I	+	+	-	+	yes
HROC183	59/f	ascending colon	G3T3N2M0	IIIb	-	+	+	+	yes

f–female, m–male, B-LCL–B lymphoid cell line, +–positive,—–negative

### *In vitro* growth kinetics and Matrigel® invasion assay

Population doubling times were determined by viable cells seeded into replicate 25 cm^2^ flasks (each 0.5 x 10^6^ cells) and daily counted for five consecutive days. Cellular invasiveness was examined using a Matrigel®-based boyden chamber assay as described [12].

### Flow cytometry and cytokine secretion pattern

**Fig 1:**
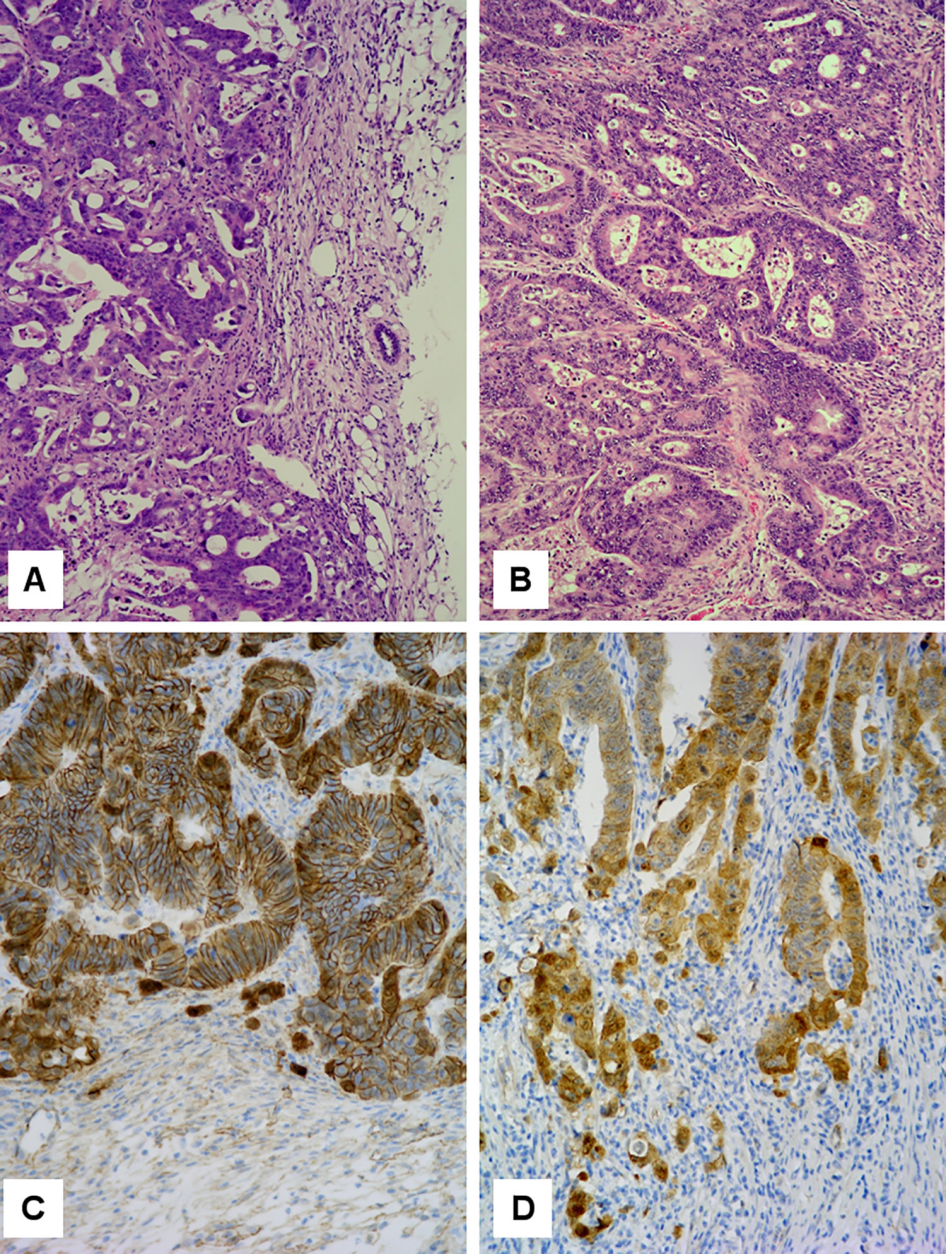
Tumor histology. H & E sections of (**A**) HROC40-PDX and (**B**) its primary. Note the invasive edge towards the right. Principal morphological features are retained in the PDX. β-catenin immunohistochemistry of (**C**) HROC60-PDX and (**D**) its primary. Note nuclear β-catenin translocation at the invasive edge of both tumors.

Surface marker expression was done by flow cytometry with and without IFN-γ (200 IU/mL for 48 hours) pre-treatment using a panel of Abs (for details see Fig 1B). Samples were analyzed using CellQuest software (BD Biosciences). Additionally, multi-color flow cytometry was done on a FACSAria using following mAbs: anti-CD47-BV421, anti-CD36-PE, anti-CD73-BV510, anti-CD95-FITC, anti-Foxp3-Alexa Fluor 647, anti-CD284-BV421, anti-CD276-PE, anti-CD133-APC, anti-cFLIP-Alexa Fluor 488, anti-Indolamin-2.3-Dioxygenase-PE, anti-BIN1 (bridging integrator 1)-Alexa Fluor 488.

Cytokine release was determined from cell free supernatants and quantified by ELISA according to the manufacturer’s instructions.

### Quality control

Quality controls included DNA Fingerprint (comparison of cell lines at different passages, matched tumor and normal tissue, as well as corresponding B cells) from genomic DNA according to [13]. Additionally, cell cultures were checked for contaminating mycoplasma and human viruses (SV40, JC/BK) [12].

### *In vitro* chemosensitivity

Triplicate wells were exposed to increasing drug concentrations (pharmacy of the University hospital Rostock). IC_50_ values of selected drugs were determined. In selected experiments, drug combinations with Azacytidine (5-Aza-2’-deoxycytidine; 30μM) were used. Doses applied in this setting were below the IC_50_ value, as tested before. Cells received two or four treatment cycles, a total of 7 and 14 days, respectively. Drug response was calculated upon crystal violet staining and measurement at 570 nm (reference wavelength: 620 nm).

### Statistics

Values are reported as the mean ± SD. IC_50_ values were calculated using Sigma Plot 12.5 software (Systat Software Inc., San Jose, CA) applying the four parameter logistic function standard curve analysis for dose response. Values are given as absolute numbers.

## Results

### Tumor histology

Primary tumors were moderately well differentiated (HROCs 40, 43, 60) or poorly differentiated (HROC183) tubular adenocarcinomas. Principal architectural and cytological features of the primaries were retained in the subcutaneous patient-derived xenografts (PDX; see Fig 1A and 1B for an example). Nuclear β-catenin translocation, if present in the primary, was also observed in the PDX (Fig 1C and 1D) and *vice versa*.

### Patient characteristics and primary cell line establishment

Cell lines were established from primary resection specimens upon surgery (clinicopathological patients’ data in Table 1). With the exception of HROC60, all tumors were resected at an advanced tumor stage (T3) showing regional lymph node infiltration, but no distant metastases. However, two out of four patients developed liver (HROC40) and brain (HROC43) metastasis, respectively, within one year after initial removal of the primary.

All cell lines were established from patients’ tumor material. Additionally, a PDX-derived cell line from HROC183 was obtained. Tumor cell cultures started to proliferative immediately upon initial culture.

Of note, *in vivo* tumorigenicity was absent in three out of four cases (positive case: HROC43, data not shown). Injecting cells at later passage (>40) and/or together with matrigel did not increase tumorigenic potential.

As determined by PCR, no contaminating mycoplasma or human pathogenic viruses (SV40, and JC/BK, data not shown) were detected within the CIMP^+^ cell lines.

### Cell morphology and phenotyping

**Fig 2:**
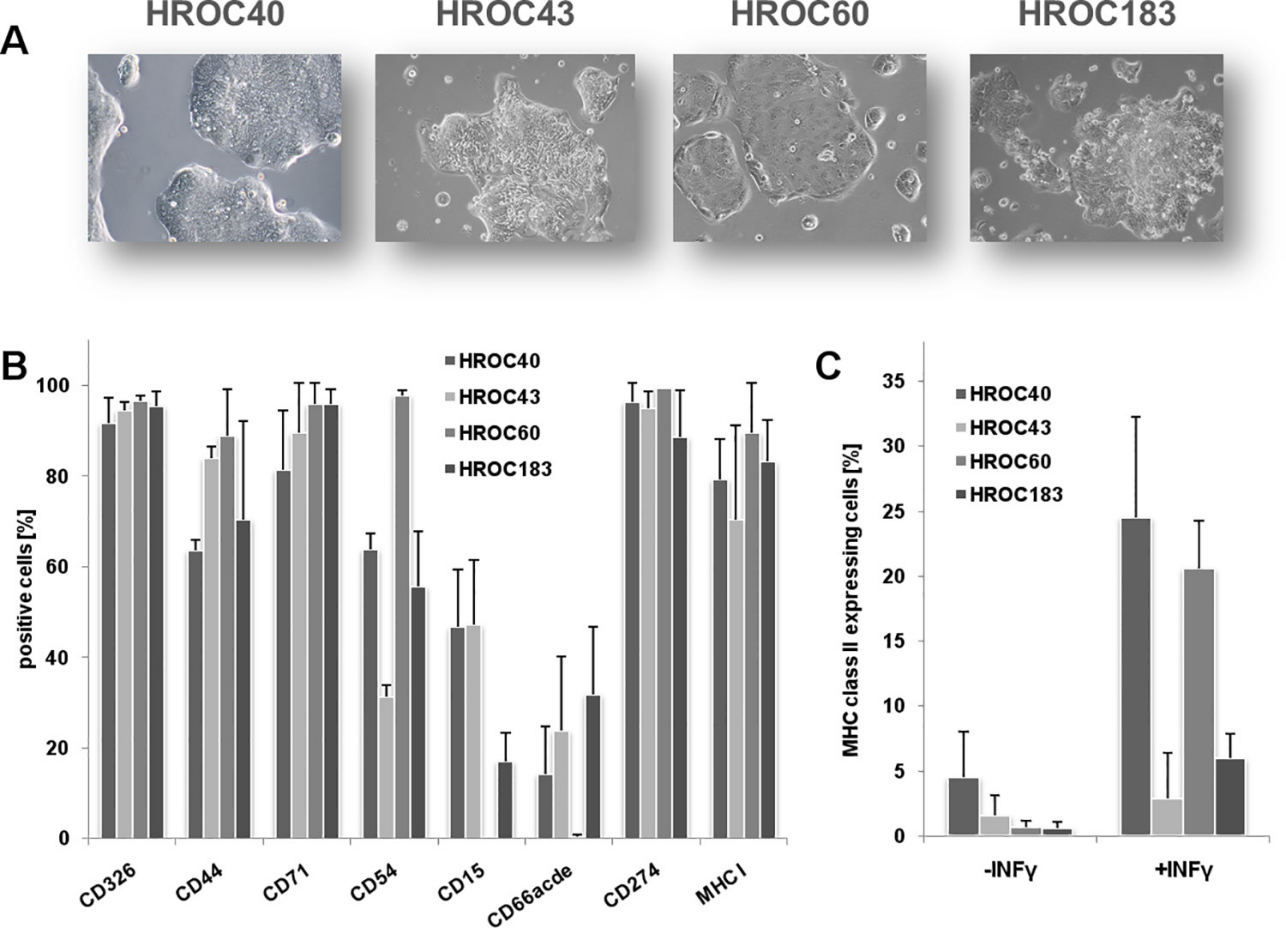
Morphology and phenotype of individual CIMP^+^ cell lines. (**A**) Light microscopy of freshly established tumor cell lines (all Passage 15). Cell lines were directly established from patients’ tumor material as described in material and methods. Original magnification ×100. (**B**) Phenotyping was conducted by flow cytometry using fluorochrome-labeled mAbs as given on the x-axis. (**C**) MHC class II expression as assessed by flow cytometry with and without IFN-γ (200 IU/mL for 48 hours) pre-treatment. Results are given as the mean % of positive cells + standard deviation of three independent experiments.

Light microscopy revealed tight adherence to the bottom of the flasks (Fig 2A). In the early cell culture (< 3 passages), several different epithelial-like cellular clones were observed. At later passage, one cell clone dominated the culture in all cases. Morphologically, all cultures appeared as multi-cellular islands forming aggregates or polygonal cell clusters. All but one of the cell cultures were dominated by a phenotypically small cell clone. The exception is HROC60. In this cell line, flat-shaped adhesive cells with large nuclei, having an irregular size and silhouette were present. HROC60 cells were the only one that grew to complete confluence.

**Fig 3:**
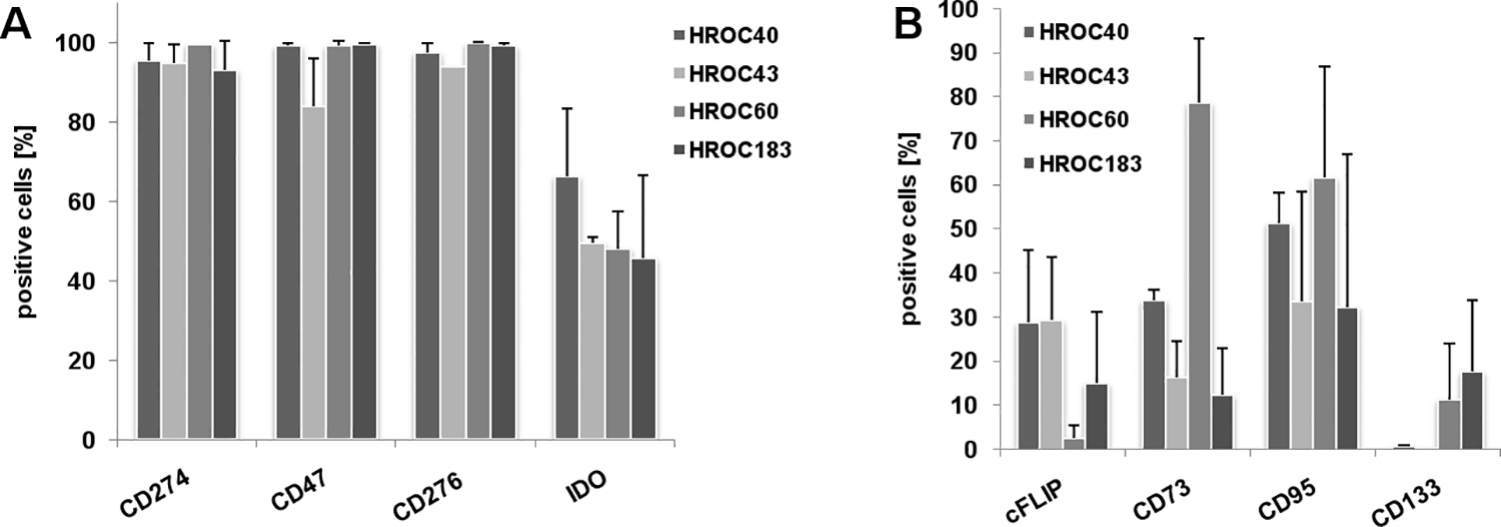
Immunosuppressive phenotype of CIMP^+^ cell lines. Assessment of immune evasion markers was conducted by multi-color flow cytometry using fluorochrom-labeled mAbs as given on the x-axis. Results are given as the mean % of positive cells + standard deviation of three independent experiments.

Additional flow cytometric phenotyping confirmed their epithelial origin (>90% EpCAM^+^). Detailed information on cellular phenotype is given in Fig 2B and 2C. In line with their immunosuppressive phenotype, several molecules, known to be linked to cancer progression and immune evasion, were highly expressed (>80%: CD47, CD274, CD276, and Indoleamine 2,3 dioxygenase-1). By contrast, an individual profile was seen for cFLIP (cellular FLICE (FADD-like IL-1β-converting enzyme)-inhibitory protein), CD73, CD95 and CD133 (Fig 3).

### Molecular features

The identity with the patient was verified by DNA fingerprint. Additional comprehensive molecular classification of cell lines was paralleled by analyses on original tumor material. Analyses identified differences in the KRAS gene mutational status between HROC60 tumor and the corresponding cell line. In line with the dynamic molecular changes, these cells acquired a c.176C>G (A59G) mutation in codon 13 of exon 2 during *in vitro* culture. However, no further differences between original tumors and corresponding cell lines were evident. Data presented in Tables 2 and 3 therefore refer to the cell lines only.

**Table 2:** Mutational profile of CIMP^+^ cell lines.

	Mutation										
Cell line	TP53				APC		KRAS		BRAF	PIK3CA	
(HROC…)	ex5	ex6	ex7	ex8	cd1	cd2	cd12	cd13	V600E	ex9	ex20
40	wt	wt	wt	mut	wt	wt	wt	mut	wt	wt	wt
43	wt	wt	mut	wt	wt	mut	mut	wt	wt	wt	wt
60	wt	wt	wt	mut	mut	wt	wt	mut	wt	wt	wt
183	wt	wt	wt	mut	mut	wt	mut	wt	wt	wt	wt

wt–wildtype, mut–mutated, ex–exon, cd–codon.

**Table 3:** Methylation marker and molecular classification of CIMP^+^ cell lines.

	DNA Methylation									
HROC…	MLH1	CDKN2A	NEUROG1	CRABP1	CACNA1G	MGMT	IGF2	SOCS2	RUNX3	Molecular type
**40**	-	+	+	+	-	-	-	-	-	**CIMP-L**
**43**	-	+	+	+	-	-	-	-	+	**CIMP-L**
**60**	-	+	+	+	+	-	-	-	-	**CIMP-L**
**183**	-	+	+	+	+	-	+	-	+	**CIMP-H**

+–methylated;—–not methylated.

**Fig 4:**
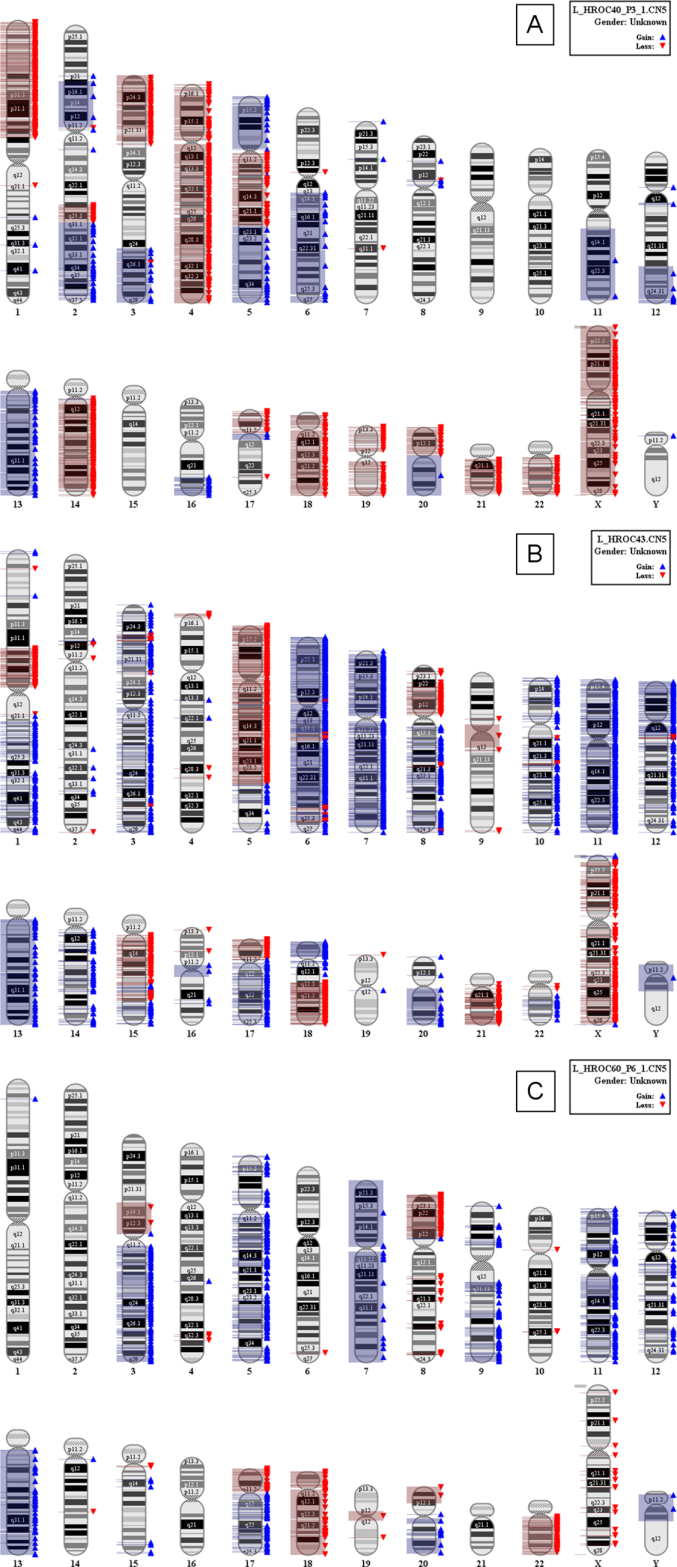
SNP Array 6.0 for assessment of CIN in CIMP^+^ cell lines. Analysis was performed according to manufacturer’s instructions. Data shown here result from three out of four CIMP^+^ cell lines. (**A**) HROC40 cells, (**B**) HROC43 cells, and (**C**) HROC60 cells. SNP Array on HROC183 did not yield evaluable data due to quality control failure.

A consensus panel for determining cytosine methylation at promoter CpG islands of tumor suppressor genes is only starting to take shape. We decided to use a combined panel covering eight tumor-specific methylation markers [16–18]. Hence, three cell lines (HROC40 (3/8 methylated promoters), HROC43 and HROC60 (each 4/8 methylated promoters)) were defined as CIMP-L and the remaining cell line HROC183 (6/8 methylated promoters) exhibited a CIMP-H phenotype. MSI was excluded by using the advanced Bethesda panel (0/6 marker). Additionally, instability on the chromosomal level was analyzed by SNP 6.0 arrays (Fig 4). Cell lines exhibited complex chromosomal aberrations, present as both deletions and insertions. Besides, typical molecular characteristics associated with the CIMP phenotype were present, i.e. KRAS mutations (codon 12 or 13 of exon 2 [19]), and BRAF^V600^ wildtype. By contrast, conflicting results have been reported for the TP53 gene [8, 20]. Here, mutations were evident in all four cases.

### Cytokine secretion profile

**Fig 5:**
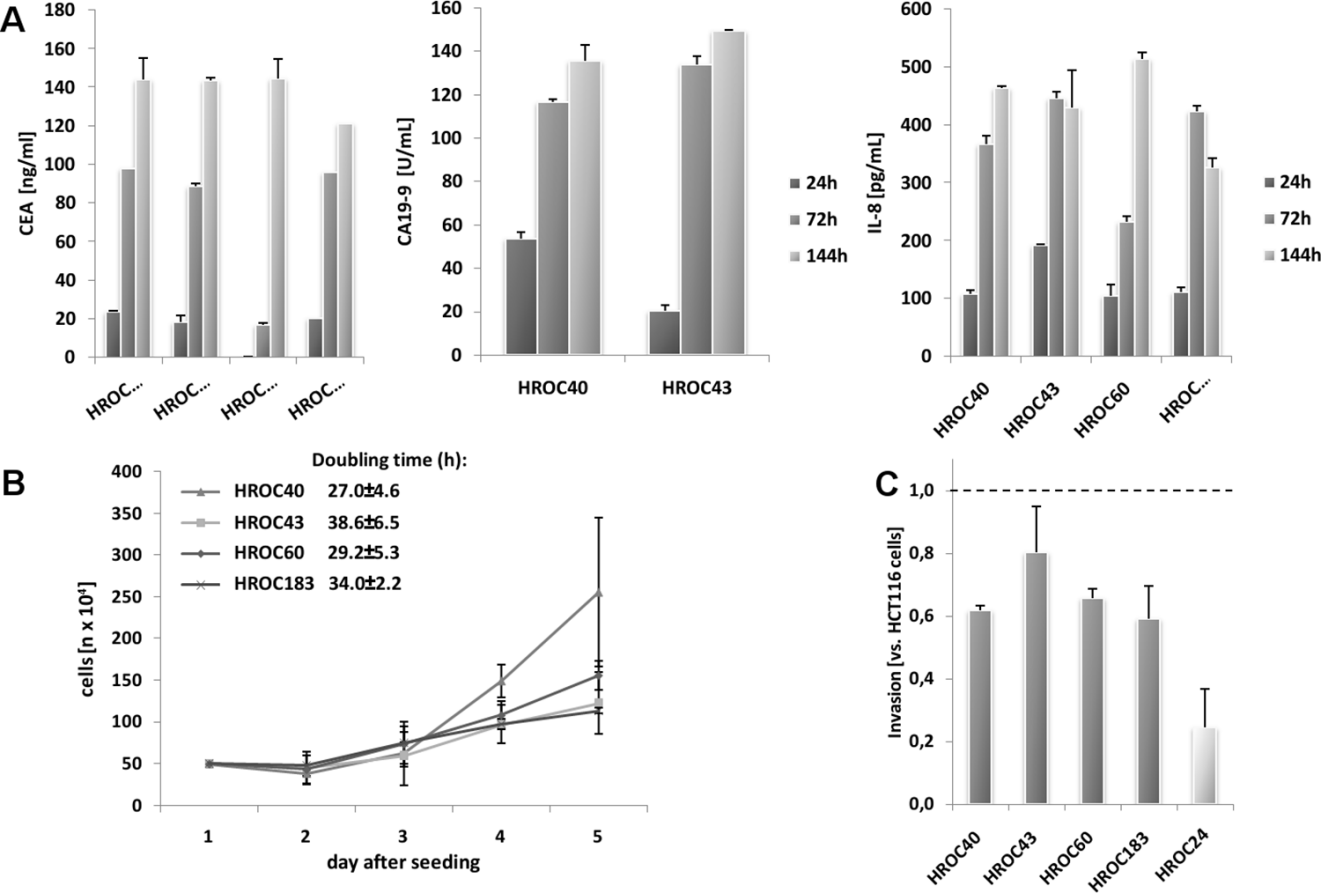
Secretion profile, growth kinetics and invasive potential of CIMP^+^ cell lines. (**A**) Cytokine secretion pattern was quantified by ELISA. Cytokine concentrations were determined by comparison with a standard curve generated from serial dilutions of individual standards. Quantitative analysis of CEA, CA19-9 and IL8 secretion after one, three and six days of culture, respectively. (**B**) Growth kinetics of cells, counted every 24 hours for five consecutive days using a Neubauer chamber. (**C**) Cellular invasiveness was examined using a Matrigel®-based Boyden chamber assay. Quantification of cellular invasiveness was estimated by MTT assay. Data are expressed as percentage invasion versus HCT116 cells (= internal positive control). (**A-C**) Results show the mean + standard deviation of three independent experiments.

All cell lines secreted high amounts of CEA in a time-dependent manner (Fig 5A). A comparable pattern was observed for CA19-9. However, this tumor marker was only detectable in supernatants of HROC40 and HROC43 cells (Fig 5A). By contrast, IL-8, an autocrine growth factor, was secreted by all four CIMP^+^ lines (Fig 5A). None of the cell lines secreted detectable amounts of IL-6, IL-10, TGF-β, or TNF-α.

### Growth kinetics and invasive potential

As anticipated, growth kinetics were quite different between cells, with HROC40 and HROC60 growing more rapidly than HROC43 and HROC183 cells (Fig 5B). Apart from these findings, we observed marginal differences with regard to their invasive potential (Fig 5C). All cell lines were less invasive than the control cell line HCT116. Highest invasion potential was seen for HROC43 cells, while the remaining three lines exhibited a comparable invasion pattern.

### Response towards classical and novel drugs

To identify potential intrinsic resistance mechanisms, a panel of clinically approved and more experimental targeted agents was applied in a 2-D *in vitro* culture system (Table 4).

**Table 4:** Drug response of CIMP^+^ cell lines.

	IC_50_								
HROC…	5-FU [μM]	Irinotecan [μM]	Cisplatin [μM]	Gemcitabine [nM]	Taxol [nM]	Erlotinib [μM]	Nilotinib [μM]	Rapamycin [μM]	Azycytidine [μM]
**40**	16	25.5	9.3	43	27.6	resistant	6.3	0.3	>30
**43**	1.5	4.5	2.7	9	45	62.5	6.9	0.2	>30
**60**	6.2	2.9	6.3	6.7	1.3	7.8	7.5	1.6	resistant
**183**	3.1	11.2	3.3	17.4	resistant	28	7	0.2	>30

Drug response was individual between cells, with HROC40 being more resistant than the remaining three lines, especially towards Irinotecan (IC_50_ = 25.5 μM). All lines were susceptible towards 5-FU, although conflicting results have been reported in the literature for this substance [9, 10]. A good response was seen towards Gemcitabine, the tyrosine kinase inhibitor Nilotinib, and Rapamycin. Erlotinib did not prove to be effective against the cell lines tested here.

**Fig 6:**
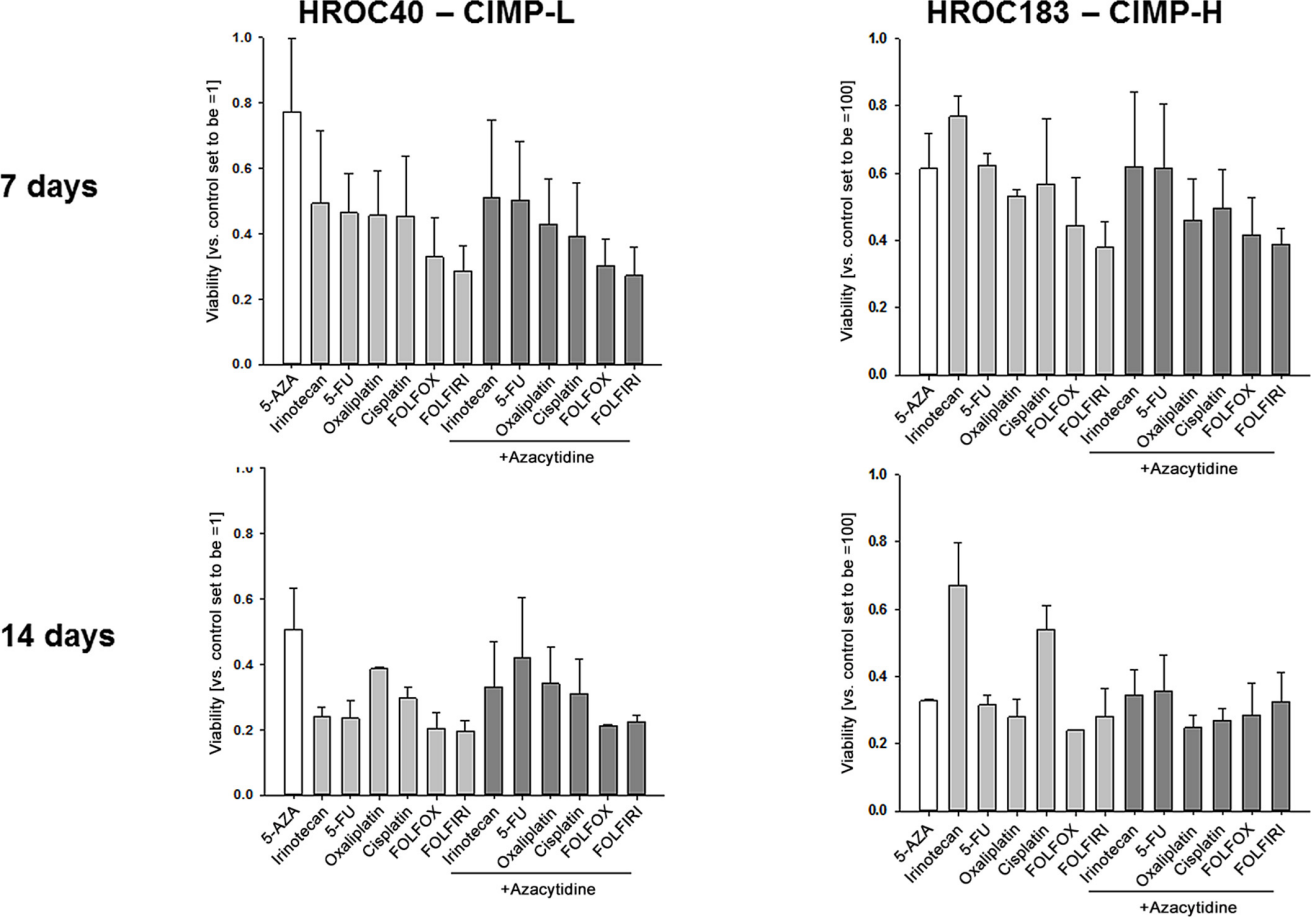
Response to Aza-based drug combinations. Representative quantitative analysis of HROC40 (left graph) and HROC183 cells (right graph) treated with Aza, standard drugs or combinations thereof. Cells received two (upper panel) or four (lower panel) treatment cycles in concentrations as indicated in the material and methods section. Cytotoxicity was quantified upon crystal violet staining and measurement at 570 nm (reference wavelength: 620 nm). Results show the mean + standard deviation of three independent experiments.

Additionally, Azacytidine alone and in combination with chemotherapeutic drugs was studied. Here, IC_50_ values for Azacytidine were above 30 μM, even in the CIMP-H cell line HROC183. Longer incubation time increased the antitumoral effect of Azacytidine (Fig 6, lower panel). Best effects were seen in HROC183 cells. Drug combinations, either given simultaneous or metronomic, slightly increased the antitumoral effect of Azacytidine (representative data are given in Fig 6). However, the different drug combinations were not better than single drug treatments and in virtually all cases; even antagonistic effects were observed (e.g. Azacytidine + 5-FU or Irinotecan; Fig 6).

## Discussion

To realize the idea of personalized medicine, we here report establishment of novel patient-individual tumor models showing molecular features of CIMP. This subtype is associated with opposed clinical performance; a good prognosis in terms of positive MSI status, while poor response and short overall survival when patients tumors are MSI-negative. For the latter, an aggressive tumor phenotype with tendency to early spread to distant organs and frequent chemoresistance has been demonstrated [5].

Though no consensus regarding classification of CIMP exists yet, we decided to combine generally accepted gene panels and marker thresholds [16–18]. Hence, three cases were classified as CIMP-L (HROC40, HROC43, and HROC60) and the remaining cell line HROC183 was CIMP-H. The latter represents a very rare subtype, since this phenotype is usually associated with MSI. Here, MSI was not present in any case. Similar genes were affected by DNA hypermethylation (i.e. CDKN2A, NEUROG1, and CRABP1). The identified RUNX3 methylation in HROC43 und HROC183 confirms an advanced tumor stage. In line with the recent literature, MGMT promoter methylation was absent, while KRAS exon 2 and TP53 gene mutations were present [8]. The exclusively high number of KRAS mutations in CIMP-associated tumors has been explained as result of a favorable selection in the specific CIMP-created environment, rather than a driving event of CIMP tumorigenesis [8]. Indeed, abnormal DNA hypermethylation is already detectable in aberrant crypt foci, the earliest lesions in the colonic mucosa [3, 8].

Cell lines generated in this study were directly obtained from parental tumor material. Additionally, PDX could be established in three out of four cases (not for HROC43). These PDX models, showing high morphologic and molecular similarity with the corresponding patient tumor, can subsequently be applied in pharmacologic studies to predict clinical response [21]. Prior to applying cost and time consuming PDX models, research is usually being performed in cell culture systems. In the early 90s, the NCI panel, including 60 cell lines, has been formulated for such studies [22]. This panel includes only a limited number of lines for any given cancer and it does not consider patient-individual differences [23]. In case of CIMP, only few cell lines have been made commercially available [24]. The accumulation of further mutations and chromosomal aberrations are further drawbacks of cultured cells. Depending on the individual tumor case and stage at resection (early vs. advanced), molecular changes cells undergo during *in vitro* culture are already present after few passages. Here, HROC60 cells acquired a KRAS mutation that was not detectable in the parental tumor. This mutation most likely reflects the rapid dynamic molecular changes that occur in tumor cells following high numbers of cell divisions [25–27]. Alternatively, considering the genetic heterogeneity within tumors, these cells may represent a single mutated clone that had not been recognized in the resection specimen of parental tumor but gave rise to *in vitro* growth. However, the fact that this cell line had been established from a very early stage tumor (UICC stage I), rather supports the first hypothesis and underlines the necessity of using ultra-low passage cells, especially for preclinical drug screening and evaluation (in especially when targeting the Ras/Raf pathway).

These freshly established cell lines were of epithelial (EpCAM^+^) origin, showing distinct morphology and growth kinetics. In line with their immunosuppressive phenotype, several molecules know to be related to immune evasion [28], apoptosis inhibition (cFLIP), tissue invasion (CD73), metastasis and consequently poor outcome (CD274) were found to be overexpressed. Moreover, all cell lines secreted high amounts of IL8 that has been linked to metastatic spread, as well [29, 30].

Patient-individual tumor models not only aid evaluating the efficacy of therapeutic strategies prior to therapy, but also help investigating resistance mechanisms of cancer cells–a major clinical problem. Here, intrinsic resistance of individual cases was detected against Erlotinib (HROC40) and Taxol (HROC183). Emphasizing the need for personalized medicine, an individual response was obtained for the commonly used antineoplastic drugs 5-FU, Irinotecan, Cisplatin, and Gemcitabine. Interestingly and in concert with our recent observation on CIN^+^/non-CIMP-associated tumors, we here observed a good response towards the mTOR inhibitor Rapamycin [13, 31]. On a molecular level, PIK3CA and/or TP53 gene mutations seem to correlate with sensitivity [32] and in fact, all CIMP^+^ cell lines described here carry a TP53 mutation. Cai and colleagues only recently reported activity against CRC stem-like cells, providing another rationale for mTOR-inhibitor tailored regimens [33].

All CIMP^+^ cell lines were responsive to the tyrosine kinase inhibitor Nilotinib [34, 35]. In the only prior study on CRC lines, a comparable growth inhibiting potential of Nilotinib could be attributed to collagen receptor targeting [36].

In summary, our novel patient-individual tumor models (cell lines + PDX) of a rare and very aggressive CRC subtype represent ideal tools to realize personalized medicine shortly. Additionally to identifying (novel) molecular target structures, drug efficacy screening and resistance mechanism identification can be done.

## Conclusion

Patient-derived tumor models provide ideal tools for identification of novel biomarkers and defining intrinsic resistance mechanisms. By combining *in vitro* and *in vivo* approaches, accurate prediction of drug responses can be realized.

## Abbreviations

CIN: chromosomal instable
CIMP: CpG island and methylator phenotype
CRC: colorectal carcinoma
MSI: Microsatellite instable
